# Dental Chemistry and Metallurgy

**Published:** 1906-08

**Authors:** Samuel S. Curry

**Affiliations:** Tarpon Springs, Fla.


### "DENTAL CHEMISTRY AND METALLURGY."

SAMUEL S. CURRY, D. D. S. Tarpon Springs, Fla.

(Florida State Dental Society, 1906.)

This is a subject that is very hard for the best of us to handle, and I hope you will pardon the mistakes that I make, for I only make a mistake when I undertake to write on this subject at all.

Chemistry in itself is a life long study, and how few of us ever find time enough to delve into the mysteries of it, when we have to make our bread out of the profession; of course we all pay attention to the chemical action of the saliva on the different filling materials, and prescribe to correct this as the case comes before us, but outside of this our chemical part of the study is neglected, for the more important parts of the practice. I might say right here that our knowledge of chemistry comes in knowing what to prescribe to build up soft teeth to harden them and every case will not answer to the same treatment, for all of our temperaments are different, so we have to consult our physicians and between the two the proper drug and diet are prescribed, and nature the great *chemist* comes to our assistance, and helps to build up what disease and neglect tears down.

Now I come to the metallurgy part of the subject, the part we all like and yet know so very little about, for in this day, with the dental depots so convenient to all, we scarcely give this line any thought, but go or send for our alloy, foil, plate or solder and we get it, and take the manufacturer's word for the karat, and as far as I know it is correct, for they should be so or the Dentists all over the country would be sending in complaints. Of course we all have our favorite makes. I have, and while other makes are just as good, I like the ones that I am used to working with.

I suppose a good many of us melt our scraps, and either use them in cast bridges or roll them into plate. I use mine in both of these ways, as I can get more out of them than to sell them to the depots to be melted over and sold back to us, in the shape of plate, and then if we are careful we will always have pure gold enough to keep the karat about the same as at first, for in the scraps there is always some small amount of solder that would lower the karet unless we added the pure gold to balance.

